# Active Individual Nanoresonators Optimized for Lasing and Spasing Operation

**DOI:** 10.3390/nano11051322

**Published:** 2021-05-17

**Authors:** András Szenes, Dávid Vass, Balázs Bánhelyi, Mária Csete

**Affiliations:** 1 Department of Optics and Quantum Electronics, University of Szeged, Dóm tér 9, 6720 Szeged, Hungary; Szenes.Andras.Laszlo@stud.u-szeged.hu (A.S.); Vass.David.Imre@stud.u-szeged.hu (D.V.); 2 Department of Computational Optimization, University of Szeged, Árpád tér 2, 6720 Szeged, Hungary; banhelyi@inf.u-szeged.hu

**Keywords:** plasmonic nanoresonator, optimization, near-field enhancement, stimulated emission enhancement, redistribution of far-field outcoupling, linewidth narrowing, mode competition, nanolaser, spaser

## Abstract

Plasmonic nanoresonators consisting of a gold nanorod and a spherical silica core and gold shell, both coated with a gain layer, were optimized to maximize the stimulated emission in the near-field (NF-c-type) and the outcoupling into the far-field (FF-c-type) and to enter into the spasing operation region (NF-c*-type). It was shown that in the case of a moderate dye concentration, the nanorod has more advantages: smaller lasing threshold and larger slope efficiency and larger achieved intensities in the near-field in addition to FF-c-type systems’ smaller gain and outflow threshold, earlier dip-to-peak switching in the spectrum and slightly larger far-field outcoupling efficiency. However, the near-field (far-field) bandwidth is smaller for NF-c-type (FF-c-type) core–shell nanoresonators. In the case of a larger dye concentration (NF-c*-type), although the slope efficiency and near-field intensity remain larger for the nanorod, the core–shell nanoresonator is more advantageous, considering the smaller lasing, outflow, absorption and extinction cross-section thresholds and near-field bandwidth as well as the significantly larger internal and external quantum efficiencies. It was also shown that the strong-coupling of time-competing plasmonic modes accompanies the transition from lasing to spasing occurring, when the extinction cross-section crosses zero. As a result of the most efficient enhancement in the forward direction, the most uniform far-field distribution was achieved.

## 1. Introduction

Different types of metal nanoresonators are capable of supporting localized surface plasmon resonances (LSPRs). Plasmonic nanorods support longitudinal and transversal resonances that can be excited via **E**-fields oscillating parallel to the longer and shorter axes, respectively [1]. Dielectric–metal core–shell particles also have a great potential to enhance the near-field due to the plasmon hybridization phenomenon [2]. The resonance wavelength can be tuned through a wide band by varying core–shell composition and generalized aspect ratio (GAR) [3]. The near-field enhancement inside such closed nanoresonators can be described analytically [4]. Due to improvement of the local density of states accompanying the near-field enhancement, plasmonic nanoresonators can prohibit or even promote the spontaneous emission of nearby fluorescent emitters [5]. The achievable near-field confinement and far-field antenna effects depend on the proper tuning of the plasmonic mode, i.e., on the design of the coupled emitter–particle configuration [6,7].

Plasmonic nanoresonators were also used to develop nanolasers that can deliver energy to the nanoscale on a femtosecond timescale. Nanolasers make it possible to achieve extremely high intensities in the electromagnetic near-field, which is important in high-intensity laser physics as well as in intracavity spectroscopy [8,9,10,11].

A seminal paper adopted the macroscopic criteria to nanolasers and determined analytical expressions for the nanolasing threshold and slope efficiency, and the existence of an optimal concentration and Purcell factor for the low threshold was shown. The increase in the concentration and Purcell factor is advantageous to delay bleaching, and larger confinement is advantageous both for lasing and bleaching thresholds [12].

To demonstrate the canonical lasing phenomena in plasmonic systems, novel numerical approaches were developed. Steady-state simulation allowed distinguishing three regions, where the gain material integrated into a plasmonic crystal behaves as an absorber, optical amplifier or a laser. To extract the optical response of the system, the coupling between the **E**-field and the population induced polarization was formulated. The effect of the **E**-field intensity on the population inversion was taken into account through a coupling term, and the gain medium was described by a complex permittivity including the local absorption and gain coefficients [13]. The spatio-temporal modeling of lasing in time-domain generalization of finite element method (FEM) was based on the solution of the wave equation formulated for the vector potential, which includes the time-dependent polarization corresponding to stimulated absorption and emission. It was shown that the lasing threshold depends on the parameters of the gain medium and the plasmon resonance promoting the enhancement, which can be optimized [14]. The Maxwell-Bloch Langevin time-domain equations were also solved, which include a noise term played as a seed aiding the transient build-up of the coherent lasing fields through feedback. It was demonstrated that above the threshold oscillations develop in time, and linewidth narrowing occurs in frequency, whereas below the threshold, only amplified spontaneous emission (ASE) is achievable [15].

Time-domain simulation of nanolaser dynamics can be a resource- and time-consuming process because of the ps–ns timescales of the population transitions. To simulate spontaneous emission, an artificial source was introduced by placing random phase dipoles at FDTD mesh points, whereas the rate equation modeling of the stimulated emission was realized by considering the slowly varying saturated state as an initial state. In this way, it was proven that both emission pathways can be enhanced when the emission frequency is coincident with the plasmonic resonance [16].

Loss compensation via metamaterials was also investigated by determining the steady-state occupations via continuous wave (CW) excitation at the absorption and by solving the time-dependent rate equations with numerical pump–probe simulations. By considering that the spatially inhomogeneous gain depends on the local pump **E**-field, it was shown that loss compensation is achieved by a good overlap between the emission and plasmonic band. To overcome radiative and dissipative losses and to reach lasing, a higher dye concentration was proposed [17]. In case of coexistent bright and dark modes, the lasing of a bright mode was demonstrated, when the high-Q dark mode was distinguished spectrally and spatially. It was shown that by changing the spectral alignment of the emission line or the spatial deposition of the gain, the relative intensities of the modes and the limits, where either one switches off, can be manipulated [18,19]. A more accurate description was achieved with frequency-domain FDTD simulations by incorporating the local **E**-field-dependent absorption at the pump wavelength and the inhomogeneous gain distribution at the probe wavelength as well [20]. The possibility of lasing improvement by plasmonically enhancing the pump phenomenon was also demonstrated [21].

A novel spasing phenomenon was discovered, where the plasmonic modes are involved in stimulated emissions. The signatures of laser action, such as the appearance of a threshold and linewidth narrowing, were experimentally demonstrated in a simple plasmonic lasing system based on a gold nanosphere coated with a dielectric layer containing dye molecules [22]. It was shown that in the case of gain-shell coated metal nanosphere-type nanolasers, the high threshold is caused by the interband transition of gold near the emission, which can be decreased by optimizing the lasing wavelength and the nanosystem geometry and by increasing the background index [23].

Plasmonic loss compensated by optical gain has been reported in the case of a nanorod coated with a dye-doped dielectric layer. It was shown that the emission wavelength can be tuned by changing the doping level and chemical composition. By increasing the dye concentration, the emission wavelength red-shifts, the threshold becomes larger and the intensity enhancement becomes smaller because of increasing losses and larger attenuation of the pump pulse. In the presence of a nanorod, more significant linewidth narrowing was observed than with a passive dielectric coating only; in addition, an ultra-small mode volume and an ultra-high Purcell factor were achieved simultaneously [24].

The weak-form solution of the wave equation formulated for the vector potential enabled the dynamic examination of the nanolasing phenomenon in FEM. The seeding of the emission channel was initialized by a small probe signal. Detailed inspection of the threshold behavior (that is inversely proportional to the spatial confinement factor) and slope efficiency (that is proportional to the Q factor) revealed that an optimal nanorod geometry exists, which allows to maximize confinement both in space and time [25].

It was shown that spasing occurs at the threshold value of the frequency and gain, whereas the achieved gain depends on the local field strength accompanying the pump. In case of synchronization, a homogeneous internal field appears in core–shell nanoparticles for a vanishing external field as well. It was evidenced that spasers’ linear modeling may result in non-physical phenomena, which can be mitigated by including the saturation into the complex dielectric function [26]. For both metal–gain and gain–metal core–shell particles, it was stated that a minimum gain threshold exists, the wavelength of which depends only on the dispersion of the bounding materials. Geometry tuning makes it possible to achieve a resonance via different shape mode factors. However, the equal effectiveness of any nanoparticle shape that has the same shape mode factor “resulting in resonance at the wavelength of minimal gain” might need revision by considering the (i) slope efficiency, (ii) near-field confinement factor and (iii) far-field extraction efficiency simultaneously [27]. It was also demonstrated that in the case of a hollow spherical plasmonic nanoparticle consisting of an oblate gain-assisted core, by increasing the aspect ratio, the threshold can be decreased, and the achieved absorptance and scattering can be increased by several orders of magnitude. When the extinction becomes zero, the linewidth significantly decreases due to the high quality of superresonance for oblate cores that promotes the spasing [28]. Different geometries were compared to consider the possible advantages for near- and far-field operation. It was shown that the light extraction efficiency is larger and decays slowly in oblate spheroids, which are ideal for lasing, whereas it is lower and decreases rapidly in prolate spheroids, which are more appropriate for local electromagnetic field enhancement [29].

There is a consensus that a laser threshold criterion has to be reconsidered in the case of nanolasers. The “S” curve, which is the intensity as a function of a pump on a log–log scale, does not work, because ideal nanolasers have β = 1 (~unity stimulated rate/spontaneous rate), i.e., they can be considered as thresholdless lasers [30]. High-intensity, coherence time and special photon autocorrelation functions are important criteria to assess, whether these systems emit a laser light. This can be evidenced by examining the linewidth narrowing, a second-order autocorrelation function, which exhibits g^2^(0): 2 → 1 modification at the transition from thermal to coherent emission [31].

In the latest reviews, the main design rules of spasers were summarized as follows: the threshold of a spaser is governed by the dielectric properties and the quality factor but does not depend directly on the geometry. The thresholdless lasing corresponds to β = 1; the minimal threshold corresponds to the case, when the mode rate is much larger than the loss in gain; and the pump approximates the mode decay rate. The typical threshold is 1–100 MW/m^2^, whereas the external quantum efficiency (EQE) is ~10% [32].

Nanolasers and spasers have promising properties for application as multifunctional optical biological probes [10,11,33,34]. They do not saturate and are more resistant to photobleaching. Molecule-specific low-toxicity ultra-fast probes with a narrow spectrum and bright emission have been reported. Spasers have a great potential for photothermal cancer therapy and photoacoustic imaging [33,34]. Ultra-narrow linewidth and population depletion of spasers can be utilized in super-resolution microscopy. Ultra-narrow spatial resolution was achieved with a few nm linewidth of dye-doped layer-coated plasmonic nanoparticles in stimulated emission depletion (STED) imaging [35,36].

In the present study, the configuration of basic individual plasmonic nanoresonators, namely nanorod- and core–shell nanoparticle-based systems, was optimized to achieve stimulated emission amplification in the near-field, to outcouple lasing power into the far-field and to enter into the spasing operation region, whose threshold is defined as the zero crossing via the extinction cross-section.

## 2. Materials and Methods

Based on the literature, the frequency-domain approach is justified by the fact that, in many cases, the properties of the equilibrium, i.e., the steady-state operation, are more interesting than the transient phenomena. The advantage of this approach is that the amplifying medium is simply described by a phenomenological permittivity or refractive index with a negative imaginary part at the emission. Moreover, optimization of the geometry and configuration for any time-dependent objective function is a difficult task for nanolasers and spasers; hence, optimization in the frequency domain may be more expedient. The post-evaluation of the dynamics in the optimized nanolasing system makes it possible to consider further potential advantages such as the dominance of the transient modes that are at play.

Plasmonic nanoresonators of metal–gain and dielectric–metal–gain composition were optimized to construct plasmonic nanolasers. The former system is a gold nanorod (NR) embedded into a gain medium, whereas the latter is a spherical silica–gold core–shell (CS) nanoparticle coated with a gain layer. The gain medium was a polymer embedding rhodamine (Rh800) dye molecules that were considered as four-level emitters with an absorbing and an emitting Lorentz oscillator at 680 and 710 nm, respectively.

Numerical pump and probe experiments were performed using frequency-domain finite element (FEM) computations realized by the RF module of COMSOL Multiphysics. A three-step study was accomplished: (i) a monochromatic, continuous wave pump beam was used at the absorption wavelength of the dye to generate the steady-state population inversion at a specific pump intensity; (ii) the CW pump beam was “turned off”, and a weak CW probe beam with an order-of-magnitude smaller intensity was used to ensure the primary photons at the emission wavelength of the dye and to determine the stimulated emission enhancement; (iii) the passive plasmonic nanoresonator coated by a dielectric layer without the active dye was also inspected. The number of dye molecules on different levels as well as the near-field intensity were monitored.

The gain material was described via the usual rate equations, including two stimulated transitions as well as the radiative and non-radiative spontaneous transitions [13,15,17,20]. These rate equations include the polarization density (**P**_i_) and the local **E**-field (**E**_i_) strength at the absorption (ω_a_) and emission (ω_e_) wavelength as well,

(1) $$\frac{\partial N_{3}}{\partial t}=\frac{1}{\hbar \omega_{a}}\left(\frac{\partial P_{a}}{\partial t}+\frac{{\Delta \omega }_{a}}{2}P_{a}\right)E_{a}-\frac{N_{3}}{\tau_{32}},$$

(2) $$\frac{\partial N_{2}}{\partial t}=\frac{1}{\hbar \omega_{e}}\left(\frac{\partial P_{e}}{\partial t}+\frac{{\Delta \omega }_{e}}{2}P_{e}\right)E_{e}-\frac{N_{2}}{\tau_{21}}+\frac{N_{3}}{\tau_{32}},$$

(3) $$\frac{\partial N_{1}}{\partial t}=-\frac{1}{\hbar \omega_{e}}\left(\frac{\partial P_{e}}{\partial t}+\frac{{\Delta \omega }_{e}}{2}P_{e}\right)E_{e}-\frac{N_{1}}{\tau_{10}}+\frac{N_{2}}{\tau_{21}},$$

(4) $$\frac{\partial N_{0}}{\partial t}=-\frac{1}{\hbar \omega_{a}}\left(\frac{\partial P_{a}}{\partial t}+\frac{{\Delta \omega }_{a}}{2}P_{a}\right)E_{a}+\frac{N_{1}}{\tau_{10}}.$$

Here the time-dependent **P**_i_ polarization vector field coupling to the **E**_i_ field was determined as follows:

(5) $$\frac{\partial^{2}P_{i}}{\partial t^{2}}+{\Delta \omega }_{i}\frac{\partial P_{i}}{\partial t}+\omega_{i}^{2}P_{i}=-\sigma_{i}{\Delta N}_{i}E_{i},$$

where i = a, e refers to the absorptance and the emission frequency, respectively; τ_10_, τ_21_ and τ_32_ are the lifetimes of the transitions; Δω_a_ and Δω_e_ are the FWHM of the corresponding spectral lines; σ_a_ and σ_e_ represent the coupling constant between the polarization and electric field at the excitation and emission; ΔN_a_ = N_3_ − N_0_ and ΔN_e_ = N_2_ − N_1_ are the population differences, whose steady state values can be determined as follows [20]:

(6) $${\Delta N}_{a}=\frac{\tau_{32}\Gamma_{03}-1}{1+\left(\tau_{32}+\tau_{21}+\tau_{10}\right)\Gamma_{03}}N,$$

(7) $${\Delta N}_{e}=\frac{\left(\tau_{21}-\tau_{10}\right)\Gamma_{03}}{1+\left(\tau_{32}+\tau_{21}+\tau_{10}\right)\Gamma_{03}}N,$$

where N is the total number density, N_k_ is the number density of dye molecules on the level of k (k = 0, 1, 2, 3) and Γ_03_ = Γ_03_(**E**_local_) is the time-independent (steady-state) effective probability of the 0 level-to-3 level stimulated transition. Based on the spatially inhomogeneous population difference, the local intensity-dependent complex dielectric permittivity of the gain material at the pump and probe frequency can be defined in the following general form [20]:

(8) $$\varepsilon \left(\omega \right)={\;\varepsilon }_{\mathrm{host}}+\sum_{i}\frac{1}{\varepsilon_{0}}\frac{\sigma_{i}{\Delta N}_{i}}{\omega^{2}+j\omega \Delta \omega_{i}-\omega_{i}^{2}},$$

assuming that both the absorption and emission bands have a Lorentzian line shape. To account for the saturation and to avoid numerical artefacts, a local **E**-field-dependent nonlinear term was included into the denominator of Equation (8) as well [27,29].

The population inversion, complex dielectric permittivity and refractive index were determined as a function of the normalized external pump field of **E**_pump_/**E**_sat_ (referred to as $E_{sat}^{pump}$) and the normalized internal local field of **E**_local_/**E**_sat_. (referred to as $E_{sat}^{local}$), where the field strength corresponding to the saturation is $E_{\mathrm{sat}}=\sqrt{4\hbar \omega_{a}{\Delta \omega }_{a}/\left(\tau_{21}\sigma_{a}\right)}$, which takes on a value of 2.94 × 10^6^ V/m for the Rh800 dye. **E**_local_ is the amplitude of the enhanced **E**-field at the pump frequency within the gain medium, which depends on the external pump **E**-field amplitude and on the nanoparticle itself. The existence of double **E**-field scales is relevant according to the considerable enhancement of the local **E**-field by the plasmonic nanoresonators. The **E**-field enhancement at the pump wavelength was determined based on the relation of these two scales **E**_local_/**E**_pump_, (referred to as $E_{sat}^{local}/E_{sat}^{pump}$) (see Supplementary Materials Table S1).

The optimization of the nanoresonator geometries was realized by using two different objective functions: (a) maximal near-field enhancement, by monitoring the maximal as well as the averaged **E**-field inside the gain medium at the lasing frequency (NF-c-type nanoresonators); (b) maximal far-field enhancement at the lasing frequency, by monitoring the absorption inside the metal and the gain medium and the power outflow and its polar angle distribution (FF-c-type nanoresonators). Here, the notation “-c” indicates the active systems with the base dye concentration (N_rod&core-shell_ = 3 × 10^25^ m^−3^).

The lasing threshold (**E**_th_) and the slope efficiency were determined based on the maximal and averaged **E**-field at the lasing frequency in the gain medium as a function of the pump and local intensity. The absorptance of the metal and gain medium and the power outflow to the far-field were also inspected at the lasing frequency. These were determined as the ohmic loss integrated in the metal and gain medium and as the radiative power integrated through a closed surface around the active nanoresonator, divided by the total incoming probe power, respectively. The internal (IQE) and external (EQE) quantum efficiencies were also evaluated (Table S1). In order to prove that the stimulated emission is enhanced by the nanoresonators, the full width at half maximum (FWHM) of the spectral distribution of the averaged **E**-field and the far-field power outflow was inspected, and the linewidth narrowing was concluded based on a comparison with the FWHM of the corresponding spectral distributions in the passive systems (Figures S1–S3).

Based on the previous literature, at a specific dye concentration, two sets of geometrical parameters should always exist, which promote maximal near-field enhancement (expected in NF-c-type nanoresonators) and efficient far-field outcoupling (expected in FF-c-type nanoresonators), respectively.

However, near-field maximization does not inherently ensure that the coupled system will enter into a spasing region, where the extinction can be completely diminished and the large gain is converted into enhanced scattering [26,27,28,29]. Therefore, for the systems optimized to maximize the near-field, the effect of the gain concentration increase on the steady-state response was inspected as well (NF-c*-type nanoresonators). Here, the notation “-c*” indicates active systems, where the dye concentration is increased post-optimization (N_rod_ = 5 × 10^25^ m^−3^ for the rod and N_core-shell_ = 8 × 10^25^ m^−3^ for the core–shell). These concentrations were inspected in more detail since they correspond to the transition between operation regions.

The underlying near-field phenomena were uncovered by studying the accompanying charge and near-field distribution and by inspecting the polar angle distribution of the far-field emission as well. The NF-c-type and FF-c-type as well as the nanorod and core–shell nanoresonators were compared to consider the advantages of different systems. Finally, the effect of concentration modification on the near-field and far-field responses as well as on the nanophotonical phenomena was inspected [17,24].

## 3. Results and Discussion

**Figure 1:**
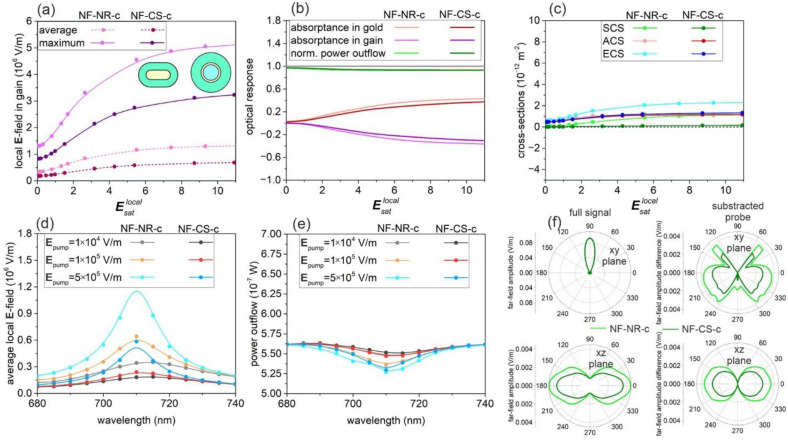
Optical response of NF-NR-c and NF-CS-c optimized nanoresonators: (**a**) Average and maximum of the enhanced local probe **E**-field in the gain medium; (**b**) absorptance in the metal nanorod and shell and in the gain medium and the normalized far-field power outflow; (**c**) optical cross-sections as a function of normalized pump **E**-field amplitude. Spectra of the (**d**) average **E**-field and (**e**) normalized power outflow at different normalized pump **E**-field amplitudes. (**f**) Polar angle distribution in the far-field at **E**_pump_ = 5 × 10^5^ V/m. Inset: geometry of NF-NR-c and NF-CS-c nanoresonators. A spline fit was added to the calculated values to guide the eyes and to facilitate the identification of trends.

**Figure 2:**
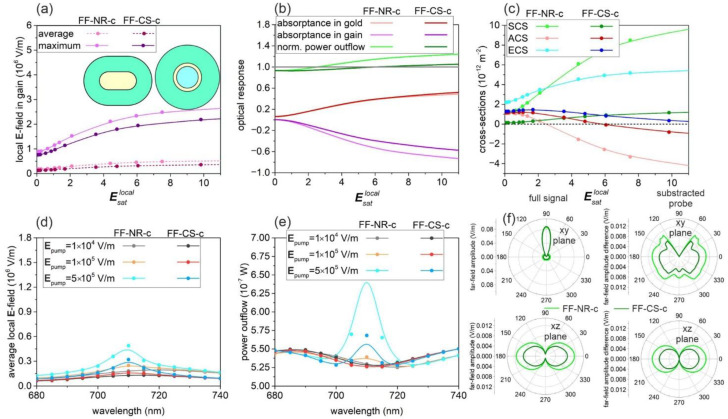
Optical responses of FF-NR-c and FF-CS-c optimized nanoresonators: (**a**) Average and maximum of the enhanced local probe **E**-field in the gain medium; (**b**) absorptance in the metal nanorod and shell and in the gain medium and the normalized far-field power outflow; (**c**) optical cross-sections as a function of normalized pump **E**-field amplitude. Spectra of the (**d**) average **E**-field and (**e**) far-field power outflow at different normalized pump **E**-field amplitudes. (**f**) Polar angle distribution in the far-field at **E**_pump_ = 5 × 10^5^ V/m. Inset: geometry of the FF-NR-c and FF-CS-c nanoresonators. A spline fit was added to the calculated values to guide the eyes and to facilitate the identification of trends.

Compared to the maximum, the average **E**-field is significantly smaller and less rapidly increases by increasing the pump intensity in both optimized systems, proving the inhomogeneity of the field distribution (Figure 1a and Figure 2a). The absorptance in the metal and gain medium has a similar “S” shape as that of the local **E**-fields (Figure 1b and Figure 2b). In both optimized active nanoresonators, the negative absorptance in the gain medium is distributed partially in the competitive loss channels of gold absorptance and the power outflow; the latter is comparable to the total power of the probe beam.

Evaluation of the optical cross-sections (OCS) makes it possible to prove the advantages of the NF-c-type and FF-c-type active nanoresonators in the achievement of large near-field enhancement and large outcoupling efficiency, respectively (Figure 1c and Figure 2c). The maximum in the near-field spectrum appears around the dye emission wavelength, which proves that the optimized systems are capable of enhancing the emission (Figure 1d and Figure 2d). The linewidth narrowing demonstrates enhanced stimulated emission, i.e., lasing-like behavior, in both of the NF-c-type and FF-c-type optimized nanoresonators (Figure 1d,e and Figure 2d,e). The pump intensity-dependent tendencies unambiguously prove that the NF-c-type nanoresonators confine the **E**-field sufficiently, whereas the FF-c-type nanoresonators couple to the far-field efficiently and also redistribute the beam spatially (Figure 1d–f and Figure 2d–f).

### 3.1. Nanorod- and Core–Shell-Mediated Near-Field Amplification: NF-c-Type Nanoresonators

According to the objective function that was defined to achieve the largest average near-field enhancement, the optimized nanorod (NF-NR-c) of a 59.38 nm long axis and 23.39 nm short axis (corresponding to aspect ratio of AR = 2.54) with a gain-coating of 18.13 nm thickness is significantly smaller in all dimensions than its FF-NR-c counterpart, which shows that it is better suited to absorb the incoming probe light than to scatter it (Figure 1a to Figure 2a insets and Table S1). The ratio of the internal local and external pump fields ($E_{sat}^{local}/E_{sat}^{pump}$) at the population saturation reveals an ~54-fold near-field enhancement (Figure S1a, Table S1). The dielectric–metal–gain multilayer composition optimization with the same objective function resulted in a core of 18.99 nm radius, a metal shell of 5 nm and a gain medium of 24.62 nm thickness in NF-CS-c. All are significantly smaller than the corresponding parameters in the FF-CS-c counterpart (Figure 1a to Figure 2a insets and Table S1). The ratio of the local and pump fields ($E_{sat}^{local}/E_{sat}^{pump}$) at the population saturation reveals an ~32-fold near-field enhancement (Figure S1a, Table S1). The near-field enhancement at the pump wavelength extracted from the ratio of saturation thresholds is smaller for NF-CS-c than for NF-NR-c.

The achieved gain effects on n(ω_a_), ε_imag_(ω_a_) and κ(ω_a_), were noticeable and significant with respect to the host medium at the pump wavelength, respectively (Figure S1b,d).

Already at the small pump, positive imaginary values are taken on; accordingly, there is a non-zero absorptance at the pump wavelength, which decreases and converges to zero by increasing the pump. The intervals where the n(ω_a_), ε_imag_(ω_a_) and κ(ω_a_) values are taken on are slightly larger for NF-CS-c than for NF-NR-c (Figure S1b,d and Table S1).

There is a considerable deviation in both the real and imaginary parts from the passive system permittivity and refractive index at the probe wavelength (Figure S1c,e). In the case of a weak pump, the small, positive ε_imag_(ω_e_) and κ(ω_e_) values indicate that the gain medium is still weakly absorbing, but above a certain pump threshold, the switching in sign proves that a gain is settled on; then, the imaginary parts gradually decrease. The intervals where the imaginary part of permittivity and refractive index values are taken on are slightly larger for NF-CS-c (Figure S1c,e and Table S1).

In NF-NR-c, after a transitional small slope region, the local probe **E**-fields linearly increase throughout $(E_{sat}^{local}$: 0.31–2.63) and then saturate (2.63 < $E_{sat}^{local}$) (Figure 1a, Table S1). The extrapolated lasing threshold in the internal local (external) pump **E**-field normalized to the **E**_sat_ is $E_{sat}^{local}$: 0.30 ($E_{sat}^{pump}$: 0.007). The (2.16 × 10^5^ V/m) slope in the average probe **E**-field is considerably smaller than that (8.52 × 10^5^ V/m) in the maximal probe **E**-field. In NF-CS-c, after the extrapolated threshold value of $E_{sat}^{local}$: 0.43 ($E_{sat}^{pump}$: 0.013) in the internal local (external) pump **E**-field normalized to **E**_sat_, the near-field increases linearly in the interval of ($E_{sat}^{local}$: 0.48–3.16) with a slope of 9.8 × 10^4^ V/m in the average probe **E**-field and with a slope of 4.97 × 10^5^ V/m in the maximal probe **E**-field.

In NF-CS-c, the linear region is wider and the lasing threshold is larger, whereas all of the slope efficiency and the achieved average and maximal probe **E**-field values are smaller than those in NF-NR-c. In NF-NR-c, the gain absorptance is positive at small pump rates and then becomes negative at the gain threshold value of $E_{sat}^{local}$: 0.32 ($E_{sat}^{pump}$: 0.006). In comparison, in NF-CS-c, the threshold of negative gain absorptance is $E_{sat}^{local}$: 0.31 ($E_{sat}^{pump}$: 0.01). The threshold of negative gain is slightly smaller (larger) in the internal local (external) pump **E**-field in NF-CS-c, similarly to the saturation threshold. The positive metal absorptance overrides the negative gain absorptance throughout the complete inspected pump interval; as a consequence, there is no far-field enhancement in NF-NR-c and in NF-CS-c (Figure 1b). The absorptances in NF-CS-c are slightly smaller, whereas the power outflow values are almost the same in the two optimized NF-c-type systems.

Accordingly, in both NF-c-type nanoresonators, the absorption cross-section (ACS) gradually increases and remains positive, which indicates an uncompensated loss (Figure 1c). In NF-NR-c, the gradually increasing scattering cross-section (SCS) becomes commensurate with the absorption cross-section; as a result, the extinction cross-section (ECS) increases more rapidly. In contrast, in NF-CS-c, the scattering cross-section remains very small throughout the inspected pump intensity interval; hence, the slowly and monotonously increasing absorption and extinction cross-sections are close to each other. As a result, all optical cross-sections are larger in NF-NR-c than in NF-CS-c.

By increasing the pump intensity, the peak on the spectrum of the average **E**-field around the passive resonance is slightly blue-shifted and becomes more intense, and its FWHM decreases (Figure 1d). The near-field spectra of NF-CS-c and NF-NR-c are similar but more than two-times less intense in NF-CS-c. In the case of NF-NR-c, in the near-field spectrum, there is no linewidth narrowing at small pump intensities; then, as a result of a 3.4-fold decrease, a 13.8 nm linewidth is reached. A monotonously decreasing tendency is observable in NF-CS-c; as a result, a slightly larger (3.6-fold) decrease is achieved with a slightly smaller rate from a smaller FWHM of the passive nanoresonator to a smaller 12.4 nm linewidth of the active nanoresonator (Figure 1d, Table S1, Figure S1f).

At the same time, the dip in the power outflow spectrum around the passive resonance deepens and undergoes linewidth narrowing by increasing the pump intensity for both NF-c-type nanoresonators (Figure 1e). In the case of NF-NR-c, beside the primary linewidth narrowing at the larger pump, a secondary dip appears at a smaller wavelength, which reveals the existence of coupled modes (Figure 1e, turquoise). In contrast, in the spectrum of NF-CS-c, there is still no sign of mode competition in the inspected pump interval (Figure 1e, blue). For further details, see Section 3.3. about NF-NR-c*-type systems. The peaks in the power outflow spectra of the passive systems are significantly narrower than the near-field peaks. They undergo a similar (1.4- and 1.6-fold) decrease with a smaller rate to considerably larger remaining linewidths (19 and 19.2 nm) in NF-NR and NF-CS-c active systems. This is in accordance with the fact that the NF-c-type nanoresonators are optimized to improve near-field properties (Figure 1f, Table S1, Figure S1f).

The polar diagram of far-field radiation shows that there is no radiation enhancement in the direction of probe propagation (90°). In the complementary polar angle regions, the NF-NR-c amplifies the radiation by redistributing it in four distinct directions. In comparison, the NF-CS-c enhances the radiation similarly, but with a considerably smaller intensity (Figure 1f).

### 3.2. Nanorod- and Core–Shell-Mediated Lasing: FF-c-Type Nanoresonators

In the case of the nanorod, when the optimization was performed to enhance the far-field stimulated emission (FF-NR-c), a 97.33 nm long axis and 47.39 nm short axis, corresponding to a smaller aspect ratio of 2.05, were determined, with a much thicker 41.33 nm gain layer coating. This indicates that the FF-NR-c is more sphere-like, and the significantly larger size of both axes shows that both the gold nanorod and the gain volume are an order of magnitude larger compared to the NF-NR-c (Figure 2a to Figure 1a insets and Table S1).

The dielectric–metal–gain multilayer composition optimization realized with the same objective function resulted in a larger core of 28.47 nm radius and a metal shell and a gain medium of 8.85 and 47.45 nm in thickness, respectively (FF-CS-c) (Figure 2a to Figure 1a insets and Table S1). These parameters correspond to a five-fold larger gain and two-fold larger metal volume compared to the NF-CS-c counterpart.

The larger size proves that both the FF-NR-c and the FF-CS-c are capable of promoting far-field scattering, rather than near-field enhancement.

In FF-NR-c, the population inversion exhibits a slower saturation at $E_{sat}^{local}$: 3.89 ($E_{sat}^{pump}$: 0.09), and the ratio of the internal local (external) pump **E**-field strength reveals a 43.22-fold near-field enhancement, which is smaller than in case of NF-NR-c (Figure S1a, Table S1). The $E_{sat}^{local}$: 4.11 ($E_{sat}^{pump}$: 0.11) internal local (external) pump field strength corresponding to saturation indicates a 37.36-fold near-field enhancement in the FF-CS-c system that is larger than that in NF-CS-c (Figure S1a, Table S1). Both the internal local and external pump field strength values corresponding to saturation are larger, but the near-field enhancement at the pump wavelength extracted from the ratio of saturation thresholds is smaller in the case of FF-CS-c compared to FF-NR-c. This indicates that the pump process is less promoted in the FF-CS-c nanoresonator than via FF-NR-c, similarly to counterpart NF-c-type nanoresonators.

A similar permittivity and index of refraction are achieved in FF-NR-c and FF-CS-c, after a (faster to slower and faster) slightly faster saturation (of imaginary and real parts in FF-NR-c) in FF-CS-c compared to their NF-c-type counterparts, respectively (Figure S2b,e). Only the ε_imag_(ω_a_) and ε_imag_(ω_e_) values are modified in slightly smaller and larger intervals in the FF-NR-c than in the FF-CS-c (Figure S2b,c, Table S1). The near-field and far-field efficiencies are slightly larger in FF-NR-c than in FF-CS-c.

However, there is a significant enhancement in efficiencies of the FF-c-type nanoresonators according to their different objective functions, when the volume fraction ratio of absorbing materials is also considered, especially for the FF-NR-c (Table S1).

In FF-NR-c, the $E_{sat}^{local}$: 0.32 ($E_{sat}^{pump}$: 0.007) lasing thresholds are just slightly larger, and the 6.83 × 10^4^ V/m (3.57 × 10^4^ V/m) slope efficiency in the linear region of the average (maximal) probe **E**-field is (almost) ~3-times smaller than that in NF-NR-c, indicating a weaker near-field confinement. The achieved maximal and average probe **E**-fields are approximately and more than two-times lower, respectively (Figure 2a to Figure 1a and Table S1). In FF-CS-c, the $E_{sat}^{local}$: 0.34 ($E_{sat}^{pump}$: 0.009) lasing thresholds are considerably smaller, and the 4.5 × 10^4^ V/m (2.9 × 10^5^ V/m) slope efficiency in the linear region is more than (almost) two-times smaller. The achieved maximal and average probe **E**-fields are more than and almost two-times smaller than those in NF-CS-c (Figure 2a to Figure 1a and Table S1). The FF-NR-c allows a smaller lasing threshold, larger slope efficiency and larger near-field enhancement at the probe wavelength than FF-CS-c does. However, the relation between FF-NR-c and FF-CS-c is similar to that of their NF-c-type counterparts in all these quantities.

Despite the significantly smaller near-field enhancement and the larger volume of FF-NR-c, the NF-c-type and FF-c-type optimized nanorod absorptance values are surprisingly close to each other at the emission (Figure 2b to Figure 1b). In FF-NR-c, the gain absorptance becomes negative at the gain threshold value of $E_{sat}^{local}$: 0.32 ($E_{sat}^{pump}$: 0.007), whereas in NF-CS-c, the threshold of negative gain absorptance is $E_{sat}^{local}$: 0.33 ($E_{sat}^{pump}$: 0.009). In FF-c-type nanoresonators, the gain threshold values are very similar to those in NF-c-type nanoresonators; however, the thresholds of negative gain become slightly larger in NF-CS-c, similarly to the saturation thresholds. Even if the ohmic loss is similar in the NF-c-type and FF-c-type nanorods, in FF-NR-c, the larger gain makes possible a larger power outflow than that in the passive system above the outflow threshold of $E_{sat}^{local}$ = 2.42. As a result, significant pump energy is converted into far-field radiation. In contrast, the absorptance value in FF-CS-c is more enhanced with respect to its NF-CS-c counterpart, but the larger gain promotes a power outflow enhancement at the larger $E_{sat}^{local}$ = 6.1 power outflow threshold (Figure 2b to Figure 1b, Table S1). The more rapidly increasing and significantly larger gain in FF-NR-c makes it possible to better enhance the far-field radiation; moreover, the power outflow threshold shows up at a significantly smaller pump than in FF-CS-c.

In FF-NR-c, the effect of the overcompensated absorption is observable in the pump-dependent cross-section tendency as well, since the absorption cross-section changes from positive to negative values at the ACS threshold of $E_{sat}^{local}$: 2.48, with increasing absolute values as the pump increases (Figure 2c, Table S1). However, due to the large scattering cross-section, the extinction remains positive and even monotonously increases throughout the inspected pump range, similarly to the NF-NR-c system. The larger cross-section values show that considerably more dynamic energy transitions occur in FF-NR-c than in NF-NR-c, again demonstrating a significant difference between the optimal NF-c-type and FF-c-type systems (Figure 2c to Figure 1c).

The initial cross-sections are significantly larger in FF-CS-c than in NF-CS-c. However, while the scattering cross-section monotonously increases and remains higher throughout the inspected pump interval, the absorption and extinction cross-sections exhibit a global maximum and then rapidly decrease. As a result, the absorption cross-section becomes negative, although at a considerably greater local pump field strength (ACS threshold of $E_{sat}^{local}$: 5.77) than that in FF-NR-c (Figure 2c, Table S1). In FF-CS-c, the negative absorption cross-section almost compensates the positive contribution of the scattering cross-section, and the extinction cross-section approaches zero. As a result, all optical cross-sections except the absorption cross-section are larger in NF-NR-c than in NF-CS-c. These results indicate that while the FF-NR-c system is far from the spaser operation region, the FF-CS-c approximates it close to the upper bound of the inspected pump interval (Figure 2c to Figure 1c).

By increasing the pump, gradually, a more intense near-field is reached, and on the spectrum, linewidth narrowing is observable on a slightly blue-shifting peak in both FF-NR-c and FF-CS-c (Figure 2d).

The amplitude of the near-field spectral peak around the passive resonance is significantly larger in FF-NR-c than in FF-CS-c. The near-field spectra of FF-NR-c and FF-CS-c show linewidth broadening at small pumps, and then, the FWHMs start to gradually decrease with increasing pump intensity (Figure 2d). The 6.1-fold decrease is much larger than that in NF-NR-c and results in a smaller FWHM (12 nm), despite the wider passive spectrum (Table S1, Figure S2f). In FF-CS-c, the 4.1-fold decrease is still considerably larger than that in NF-CS-c; however, the linewidth remains slightly larger (12.9 nm) at the largest inspected pump intensity (Table S1, Figure S2f). Accordingly, the linewidth of the near-field spectrum of FF-NR-c is always larger than that of FF-CS-c, except at the largest pump, where they are comparable.

The far-field spectrum of FF-NR-c and FF-CS-c exhibits a peak instead of a dip at two consecutive pump intensities and at the highest applied pump intensity, respectively (Figure 2e). While both the near-field and power outflow spectra are similar in FF-NR-c and FF-CS-c, a dip-to-peak switching in the power outflow spectrum can be seen at smaller pump values in FF-NR-c. The FWHM of the power outflow spectrum gradually and rapidly decreases in FF-NR-c; the large 8.2-fold decrease results in a 6.7 nm linewidth, which is three-times smaller than that in NF-NR-c (Figure 2e, Figure S2f). In contrast, in the case of FF-CS-c, sudden narrowing occurs at the largest pump intensity; however, due to the largest 10.9-fold decrease from an intermediate passive linewidth, the reached 3.9 nm FWHM is five-times smaller than that in NF-CS-c, which is the smallest among the inspected systems (Table S1, Figure S2f). The FF-c-type nanoresonators show a larger bandwidth compared to their NF-c-type counterparts at small pumps, but this relation is reserved at the largest inspected pump intensity, proving the advantage of these systems in far-field enhancement. The probe signal is not only amplified but it is redistributed in polar angles as well (Figure 2f). The amplification is larger in FF-c-type than in NF-c-type nanoresonators, and the redistribution is spatially more uniform. The difference between FF-NR-c and FF-CS-c intensity is relatively small compared to the NF-c-type optimization.

By comparing NF-c-type and FF-c-type nanoresonators, one can conclude that the population inversion saturation is faster in the NR and CS nanoresonators optimized to maximize near-field enhancement (Figures S1a and S2a). At the pump wavelength, the ε_real_(ω_a_) uniformly is not modified, and ε_imag_(ω_a_), as well as n(ω_a_) and κ(ω_a_), decreases slightly more rapidly in NF-c-type nanoresonators (Figures S1b and S2b,d). At the probe wavelength, ε_real_(ω_e_) and n(ω_e_) decrease more rapidly, whereas the decrease in ε_imag_(ω_e_) and κ(ω_e_) is modified, from faster to slower, in the nanoresonators optimized to maximize the near-field enhancement. In comparison, these tendencies are reversed in the nanoresonators optimized for far-field outcoupling (Figures S1c,e and S2c,e). In NF-NR-c and FF-NR-c, ε_imag_(ω_e_) and κ(ω_e_) zero crossing occurs at $E_{sat}^{local}$: 0.52 and at $E_{sat}^{local}$: 0.62, but their relation is modified at $E_{sat}^{local}$~4 (Table S1). The ε_imag_(ω_e_) and κ(ω_e_) values are smaller in NF-CS-c throughout the whole inspected interval and cross zero earlier at $E_{sat}^{local}$: 0.56, whereas in FF-CS-c, zero crossing occurs later at $E_{sat}^{local}$: 0.73.

The lasing threshold is slightly smaller in NF-NR-c than in FF-NR-c, whereas this relation is reversed in CS-c nanoresonators. The slope efficiency is uniformly larger for NF-c-type nanoresonators; as a result, the local field increases more rapidly to a larger value before saturation than in FF-c-type nanoresonators (Figure 1a to Figure 2a).

The absorptance in gold is very similar, but it is smaller in NF-c-type nanoresonators. The absorptance in the gain medium is larger throughout the complete pump interval in FF-c-type nanoresonators (Figure 1b to Figure 2b). The threshold in gain is equal for NR-c nanoresonators, whereas it is smaller in NF-CS-c than in FF-CS-c. For NF-c-type systems, the power outflow remains smaller than the total power of the probe beam throughout the complete inspected pump interval, whereas in FF-c-type nanoresonators, the outflow overrides it above a certain outflow threshold of the pump (Figure 1b to Figure 2c).

The absorption cross-section of the smaller maximal absolute value remains positive in NF-c-type nanoresonators, whereas it takes on larger absolute values and becomes negative at a certain ACS threshold in FF-c-type nanoresonators.

It is monotonous for NF-CS-c and FF-NR-c, whereas it exhibits an extremum for NF-NR-c and FF-CS-c. The scattering and the extinction cross-section exhibit a significantly slower monotonous increase to smaller values for the NF-NR-c than in the FF-NR-c. Both the scattering and extinction cross-section monotonously increase for NF-CS-c, whereas the scattering cross-section monotonously increases, but the extinction cross-section exhibits a maximum for FF-CS-c (Figure 1d to Figure 2d). The average **E**-field increase is faster, but the FWHM decreases less rapidly in NF-c-type nanoresonators (Figure 1d to Figure 2d, Figures S1f–S2f). The outflow gradually decreases by increasing the pump, and the FWHM decreases less rapidly in NF-c-type systems.

The power outflow spectrum switches from a dip to a peak above a certain threshold and the FWHM decreases more rapidly in FF-c-type nanoresonators (Figure 1e to Figure 2e, Figures S1f–S2f). The far-field lobes are larger and are less dependent on the nanoresonator composition in the case of FF-c-type nanoresonators (Figure 1f to Figure 2f).

### 3.3. Nanorod- and Core–Shell-Mediated Spasing: NF-c*-Type Nanoresonators

By increasing the concentration in the [3 × 10^25^ m^−3^, 3 × 10^26^ m^−3^] interval, the population inversion saturation behavior of NF-NR-c*-type nanoresonators is intermediate in between NF and FF-NR-c, whereas it increases and saturates most rapidly in NF-CS-c* (Figures S1a–S3a). This already indicates that NF-NR-c* is a kind of intermediate system, whereas NF-CS-c* differs from both NF and FF-CS-c systems. We have selected those concentrations that result in zero crossing of the extinction cross-section in the inspected pump interval, i.e., below the damage threshold of the nanoresonators.

In NF-NR-c*, in the case of a 5 × 10^25^ m^−3^ dye concentration, saturation occurs at $E_{sat}^{local}$: 3.67 ($E_{sat}^{pump}$: 0.08), and the comparison of the internal local and external pump **E**-field strengths reveals a 45.88-fold near-field enhancement at the pump wavelength, which is intermediate with respect to the enhancements achieved in NF-NR-c and FF-NR-c (Figure 2a, Table S1). The NF-NR-c* approximates the saturation dynamics of FF-NR-c at $E_{sat}^{local}$ = 6.

In NF-CS-c*, in the case of a 8 × 10^25^ m^−3^ dye concentration, saturation occurs at $E_{sat}^{local}$: 2.79 ($E_{sat}^{local}$: 0.11), and the comparison of the internal local and external pump **E**-field strengths reveals a 25.36-fold near-field enhancement at the pump wavelength, which is smaller than the enhancement reached in either NF-CS-c or FF-CS-c (Figure 2a, Table S1). The saturation arises at a significantly smaller $E_{sat}^{local}$ value in NF-CS-c* than in NF-NR-c*; moreover, the saturation process is faster. This relation is similar (reversed) with respect to the counterpart NF-c-type (FF-c-type) nanoresonators.

In both NF-NR-c* and NF-CS-c*, at the pump wavelength, the difference with respect to the passive system becomes significantly larger in ε_imag_(ω_a_), as well as in n(ω_a_) and κ(ω_a_) (Figures S1b,d–S3b,d, Table S1). Their values become significantly larger and their decrease is considerably faster than in either of the NF-c-type or FF-c-type counterparts. This causes larger detuning and larger absorption as well.

In both NF-NR-c* and NF-CS-c*, at the probe wavelength, the significantly larger difference in the real parts (ε_real_(ω_e_) and n(ω_e_)) results in larger detuning; however, the real parts decrease faster than in either the NF-c-type or FF-c-type nanoresonator counterpart. The more rapidly decreasing imaginary parts (ε_imag_(ω_e_) and κ(ω_e_)) result in a pronounced negative absorption, and the imaginary parts decrease through a wider interval than in either NF-c-type or FF-c-type nanoresonator counterpart (Figure S3c,e, Table S1). The values of ε_imag_(ω_a_), n(ω_a_), κ(ω_a_), ε_real_(ω_e_) and n(ω_e_) are larger, whereas ε_imag_(ω_e_) and κ(ω_e_) are smaller in NF-CS-c* than in NF-NR-c* (Figure S1b,e–S3b,e). All optical parameter intervals are wider in the case of NF-CS-c* than in NF-NR-c* (Table S1). The initial and final deviations of NF-CS-c* are larger in all values and decrease faster than in NF-NR-c* (Figure S3c,e).

In NF-NR-c*, the [$E_{sat}^{local}$: 0.65–2.07] region of linear near-field enhancement is contracted, and the largest lasing threshold values ($E_{sat}^{local}$: 0.45 ($E_{sat}^{pump}$: 0.013)) are reached with the largest (4.84 × 10^5^ V/m (1.88 × 10^6^ V/m)) slope efficiency in the average (maximal) probe **E**-field among the nanorod-based nanoresonators.

**Figure 3:**
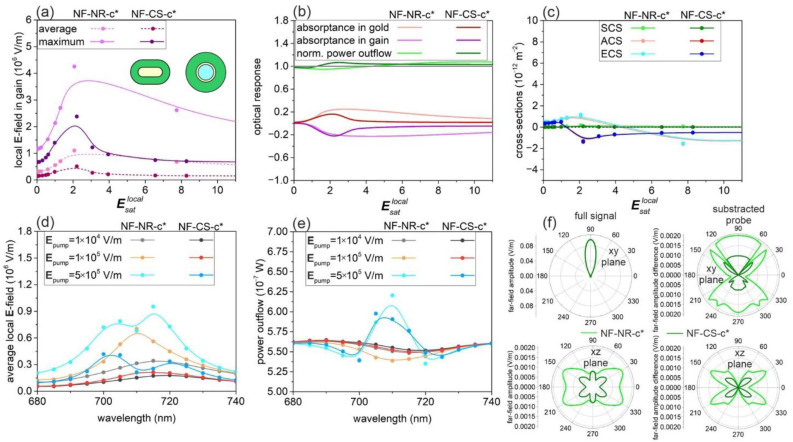
Optical response of post-optimization concentrated NF-NR-c* and NF-CS-c* nanoresonators: (**a**) Average and maximum of the enhanced local probe **E**-field in the gain medium; (**b**) absorptance in the metal nanorod and shell and in the gain medium and the normalized far-field power outflow; (**c**) optical cross-sections as a function of pump amplitude. Spectra of the (**d**) average **E**-field and (**e**) far-field power outflow at different normalized pump **E**-field amplitudes. (**f**) Polar angle distribution in the far-field at **E**_pump_ = 5 × 10^5^ V/m. Inset: geometry of NF-NR-c* and NF-CS-c* nanoresonators. A spline fit was added to the calculated values to guide the eyes and to facilitate the identification of trends.

At small and large pumps, an intermediate value of the average probe **E**-field is achieved; however, instead of saturation, a rapid monotonous near-field decrease occurs after a global maximum at $E_{sat}^{local}$ = 2.07 (Figure 3a, Table S1).

In NF-CS-c*, the [$E_{sat}^{local}$: 0.30–2.20] region of linear near-field enhancement contracts, but the smallest lasing threshold values $E_{sat}^{local}$: 0.29 ($E_{sat}^{pump}$: 0.011)) are reached with the largest (1.87 × 10^5^ V/m (8.83 × 10^5^ V/m)) slope efficiency in the average (maximal) probe **E**-field among the CS nanoresonators (Figure 3a, Table S1). At small and large pumps, the smallest average **E**-field is achieved, but similarly to the NF-NR-c*, instead of saturation, a rapid near-field decrease occurs after a global maximum at $E_{sat}^{local}$ = 2.2.

In near-field enhancement, both systems override the counterpart NF-NR-c and NF-CS-c nanoresonators at their maximum. In comparison, the lasing threshold is larger, but the slope efficiency as well as the achieved maximal and average local probe **E**-field strengths are also larger in NF-NR-c* than in NF-CS-c*. Another difference is the faster decrease in the near-field in NF-CS-c*, when the pump is further increased.

The onset of gain occurs at a gain threshold of $E_{sat}^{local}$: 0.29 ($E_{sat}^{pump}$: 0.006) in NF-NR-c* and $E_{sat}^{local}$: 0.31 ($E_{sat}^{pump}$: 0.012) in NF-CS-c*. The onset of gain occurs at smaller $E_{sat}^{pump}$ and $E_{sat}^{local}\;$values in the case of NF-NR-c*. Correspondingly, in both NF-NR-c* and NF-CS-c*, the gold absorptance has a similar (smaller) initial value compared to the counterpart NF-c-type (FF-c-type) nanoresonators, but more rapidly increases and exhibits a maximum value that overrides the absorptance in FF-c-type nanoresonators as well, and then starts to decrease.

The gain exhibits a similar tendency as the gold absorptance, but with a slightly larger absolute value, which promotes the far-field power outcoupling. The decrease is more rapid in NF-CS-c* than in NF-NR-c*; however, the reached maximal gain is larger in NF-CS-c* nanoresonator (Figure 3b).

In the far-field response, an enhanced power outflow appears, which reveals that the energy of the probe can be enhanced and outcoupled as well (Figure 3b). This is because the smaller gain is accompanied by an even smaller absorptance in the metal rod (shell), and as a result, the loss is moderated as well. However, in NF-NR-c*, this outcoupling occurs at a larger pump than in FF-NR-c, and the outcoupled power remains smaller. In contrast, in NF-CS-c*, the outcoupling arises at a smaller pump than in FF-CS-c, and its amplitude reaches larger values. In NF-NR-c* and NF-CS-c*, the initial values of power outflow are similar, but in the case of NF-CS-c*, it begins to increase earlier and faster. The outcoupling is achieved at a considerably smaller pump ($E_{sat}^{local}$ = 1.24) in NF-CS-c* than in NF-NR-c* ($E_{sat}^{local}$ = 4.29); however, the maximal outcoupled power reached in NF-CS-c* at ($E_{sat}^{local}$ = 2.2) is almost equal to that in NF-NR-c* at $E_{sat}^{local}$ = 7.7 (Figure 3b, Table S1).

Based on the pump-dependent cross-section values in both NF-NR-c* and NF-CS-c*, the scattering cross-section is relatively small and exhibits a maximum at $E_{sat}^{local}$ = 2.1 and $E_{sat}^{local}$ = 2.2. In NF-NR-c* (NF-CS-c*), the absorption cross-section also shows a maximum at $E_{sat}^{local}$ = 2.1 ($E_{sat}^{local}$ = 1.0), and then, it becomes negative at $E_{sat}^{local}$ = 4.24 (1.26) as the pump intensity increases (Figure 3c). The extinction cross-section is governed by the absorption cross-section; hence, in contrast to the NF-NR-c and NF-CS-c, it also exhibits a maximum and then becomes negative at $E_{sat}^{local}$ = 4.53 (1.29).

Both the absorption and extinction cross-sections reach their minimum at pump values corresponding to the maximal power outflow. The comparison of NF-CS-c* to NF-NR-c* shows that the scattering cross-section is similarly small, the initial absorption cross-section is also smaller in NF-CS-c*; moreover, it becomes negative more rapidly and exhibits a narrower dip of a similar negative value than that in NF-NR-c*. The extinction cross-section is analogously determined by the absorption cross-section in NF-c*-type nanoresonators.

The near-field spectra in NF-NR-c* (NF-CS-c*) indicate that the large refractive index shift at smaller pump values makes the optimized system non-resonant around the emission wavelength, i.e., the peak red-shifts to ~715 nm (~720 nm). Compared to counterpart NF-c-type and FF-c-type nanoresonators, the near-field increases most rapidly from an intermediate level and exhibits narrowing but splits at large pump intensities. In the case of very intense pumping, an additional peak emerges again around 705 nm (700 nm), with neighboring dips on the near-field spectra (Figure 3d). The only difference is that the splitting results in a global maximum at a larger (smaller) wavelength in NF-NR-c* (NF-CS-c*), and the intensity of the spectra is always lower in NF-CS-c*. In NF-NR-c* and NF-CS-c*, the linewidths of the near-field spectra are similar; however, due to the smaller 3.2-fold linewidth narrowing, the final linewidths of 14.6 and 14.3 nm remain larger compared to the values of NF-c-type counterparts (Figure 3d, Table S1, Figure S3f). This indicates that the NF-c*-type systems are not optimal in the near-field. The NF-CS-c* shows no linewidth narrowing at small pump intensities, but at the largest pump, its near-field spectral bandwidth is comparable to that of NF-NR-c*.

The far-field spectrum not only switches from a dip to a peak, but it also exhibits a narrowing; however, the FWHM decrease is slightly (significantly) slower in NF-NR-c* (NF-CS-c*) than in FF-c-type counterparts (Figure 3e). At the highest inspected pump, the maximum on the far-field spectrum is coincident with 710 nm in NF-NR-c*, whereas it is still shifted to 705 nm in NF-CS-c* and shows asymmetry as a sign of coupled modes’ co-existence. The dips in NF-NR-c* spectra are deeper and are closer, whereas the peak is more intense. In its power outflow spectrum, NF-NR-c* shows significant 1.8-fold narrowing arising only at the largest inspected pump intensity (Figure 3e, Table S1, Figure S3f). Due to the larger rate of FWHM decrease, the obtained 15.5 nm linewidth is smaller than that in NF-NR-c.

Similarly, in NF-CS-c*, the 1.9-fold decrease is larger; as a result, the reached minimal 16.3 nm linewidth is smaller than the values in the NF-CS-c counterpart ((Figure 3e, Table S1, Figure S3f). Despite the larger rate of narrowing in NF-CS-c*, the linewidth remains slightly smaller in NF-NR-c* according to the narrower passive nanoresonator bandwidth.

At a high intensity, the increased concentration becomes advantageous, but spectral bandwidths in NF-c*-type nanoresonators are intermediate with respect to the counterpart NF-c-type and FF-c-type active nanoresonators. This is in accordance with the pump intensity dependence of the power outflow, which can be enhanced with increased concentration throughout a wide band (Figure 3b).

According to the polar diagram of far-field emission, NF-NR-c* and NF-CS-c* are unique systems in the sense that they are capable of amplifying the probe beam along the direction of propagation; hence, the spatial redistribution in polar angles becomes significantly less inhomogeneous compared to NF-c-type and FF-c-type counterparts. Similar (in contrast) to the NF-c-type and FF-c-type optimized nanoresonators, based on the EQE the outcoupling into the far-field is more efficient in NF-CS-c* than in NF-NR-c*. These unique transitions indicate that by considerably changing one of the configuration parameters—the dye concentration in the present case—the optical response may undergo a significant modification as well.

**Figure 4:**
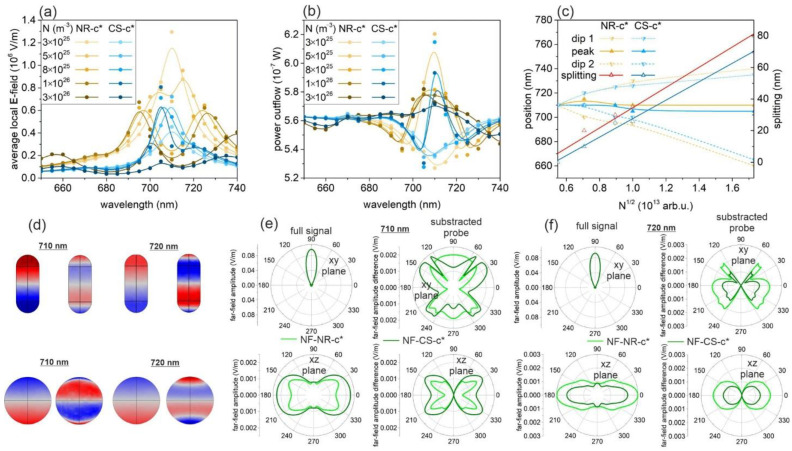
Effect of the dye concentration modification: (**a**) averaged **E**-field and (**b**) far-field power outflow spectra at different normalized pump **E**-field amplitudes. (**c**) Peak and dip positions and dip splitting as a function of dye concentration. (**d**) Charge distribution and (**e**,**f**) far-field polar diagram at the (**d**,**e**) central peak (710 nm) and (**d**,**f**) one of the dips, in the case of 5 × 10^25^ m^−3^ and 8 × 10^25^ m^−3^ dye concentration and a pump intensity of 5 × 10^5^ V/m and 3 × 10^5^ V/m for NF-NR-c* and NF-CS-c*, respectively. A spline fit was added to the calculated values to guide the eyes and to facilitate the identification of trends.

The near- and far-field spectra at the pump intensities, which result in the maximal outcoupling in NF-NR-c* and NF-CS-c*, indicate that there is a strong interaction between different co-existing modes (Figure 4a,b). Undoubtedly, the spectra collected by increasing the concentration from 3 × 10^25^ m^−3^ to 3 × 10^26^ m^−3^ reveal that at intermediate concentration (5 × 10^25^ m^−3^ and 8 × 10^25^ m^−3^), a peak appears in between two dips on the far-field spectral response of NF-NR-c* and NF-CS-c* (Figure 4a,b).

The spectral distance of these dips is proportional to the $\sqrt{N}$ at intermediate concentrations, which reveals that two modes are strongly coupled in the active nanoresonator (Figure 4c). The time evolution of the charge separations at the peak and neighboring dips has been extracted from the steady-state FEM computations, which implicitly comprises full, time-harmonic dynamic data extensions.

The time evolution proves that, although the dipolar mode is dominant at all extrema, at the peak, a quadrupolar charge separation appears in an enhanced fraction of each time cycle (Figure 4d, Supplementary Materials). However, this transient phenomenon becomes less dominant by further increasing the concentration. The symmetry of the polar angle distribution of the far-field emission pattern also indicates the co-existence of different modes (Figure 4e,f).

## 4. Conclusions

The comparison of the nanorod- and core–shell-based NF-c-type and FF-c-type nanoresonators indicates that the nanorod exhibits a smaller lasing threshold, larger slope efficiency and larger achieved **E**-field intensities due to the larger near-field enhancement. The threshold in gain is slightly larger (smaller) in NF-NR-c (FF-NR-c), the optical cross-sections are larger in NF-NR-c and FF-NR-c and the FF-NR-c exhibits a smaller threshold in power outflow and absorption cross-section than the counterpart CS-c nanoresonators. The peaks on the near-field spectra are larger, and the power outflow spectrum of NF-NR-c exhibits a sign of coupled modes.

In addition, the far-field spectrum switches from a dip to a peak at smaller pumps and exhibits larger peaks in FF-NR-c. According to these results, the nanorod-based systems could be proposed for nanolasing in the case of moderate concentrations. However, the spectral bandwidth is smaller in the near-field in NF-CS-c and in the far-field in FF-CS-c with respect to the NR counterparts. The IQE and EQE are also slightly larger in NF-CS-c but almost equal in FF-c nanoresonators. These signatures already indicate that the core–shell nanoresonators are also competitive.

In the case of post-optimization increased concentration, the slope efficiency and the achieved **E**-field intensity remain larger and the gain threshold is slightly smaller for the nanorod, but the lasing threshold becomes smaller for the core–shell nanoresonator. In addition to this, the power outflow, ACS and ECS threshold also become smaller for the NF-CS-c* nanoresonator. The near-field and far-field are typically slightly smaller, but there is a pump interval ($E_{sat}^{local}$:1.5–6) where the power outflow from NF-CS-c* is significantly larger than from NF-NR-c*. In addition, the IQE and EQE are also significantly larger in NF-CS-c*. Based on the comparison of the spectral, near-field and far-field responses at the pump intensities corresponding to power outflow maxima, at larger concentrations, the core–shell nanoresonators become more advantageous. In terms of linewidth narrowing, both NF-c*-type nanoresonators are weaker in the near-field and intermediate in the far-field. The bandwidth is relatively narrower in NF-CS-c* in the near-field and in NF-NR-c* in the far-field.

In both compositions of nanoresonators, the increase in concentration allows the transition to spasing, which requires a configuration, where the ECS crosses zero and the gain is converted into far-field power outflow. To the best of our knowledge, this is the first case where the strong-coupling of time-competing dipolar and quadrupolar modes is uncovered, and their role in facilitating the lasing-to-spasing transition is proven. A further advantage of NF-c*-type nanoresonators is that they promote the redistribution of the far-field emission.

## Data Availability

The data underlying the results presented in this paper are not publicly available at this time but may be obtained from the authors upon reasonable request.

## Supplementary Materials

The following are available online at https://www.mdpi.com/article/10.3390/nano11051322/s1, Figure S1: Pump-dependent optical properties of optimized NF-c-type nanoresonators, Figure S2: Pump-dependent optical properties of optimized FF-c-type nanoresonators, Figure S3: Pump-dependent optical properties of post-optimization concentrated NF-c*-type nanoresonators, Table S1: Optical response of optimized and post-optimization concentrated systems, Video S1: Time-dependent surface charge distribution of NF-NR-c* at 710 nm, Video S2: Time-dependent surface charge distribution of NF-NR-c* at 720 nm, Video S3: Time-dependent surface charge distribution of NF-CS-c* at 710 nm, Video S4: Time-dependent surface charge distribution of NF-CS-c* at 720 nm.
