# Supplementary material for: Active Individual Nanoresonators Optimized for Lasing and Spasing Operation
Source: Nanomaterials (Basel). 2021 May 17;11(5):1322. doi: 10.3390/nano11051322 (PMC8155829; doi:10.3390/nano11051322)
Supplement: Supplementary file 1 [file nanomaterials-11-01322-s001.zip › nanomaterials-1200821-Supplementary Materials/nanomaterials-1200821-Supplementary Materials.pdf]

# Supplementary Materials

# Active Individual Nanoresonators Optimized for Lasing and Spasing Operation

**András Szenes [1], Dávid Vass [1], Balázs Bánhelyi [2] and Mária Csete [1,*]**

[1] Department of Optics and Quantum Electronics, University of Szeged, Dóm tér 9, 6720 Szeged, Hungary; Szenes.Andras.Laszlo@stud.u-szeged.hu (A.S.);Vass.David.Imre@stud.u-szeged.hu (D.V.)

[2] Department of Computational Optimization, University of Szeged, Árpád tér 2, 6720 Szeged, Hungary; banhelyi@inf.u-szeged.hu

* Correspondence: mcsete@physx.u-szeged.hu

The population differences saturate (reach the criterion of $N_2$-$N_1$=$N_0$-$N_3$=0.5 in inversion), as the pump amplitude increases (Figure S1–3a). The saturation occurs significantly below the nominal pump saturation amplitude, which indicates a considerable near-field enhancement at the pump wavelength as well due to the nanorod (NR) and core-shell (CS) plasmonic nanoresonators. At the same time, the saturation is reached considerably above the nominal maximum near-field amplitude, which indicates a spatially inhomogeneous near-field enhancement.

The deviation with respect to the passive system becomes asymptotical in all optical quantities ($\varepsilon_{real}(\omega)$+i*$\varepsilon_{imag}(\omega)$ and n(ω)+i*κ(ω)) by increasing the pump (Figure S1–3b–e, Table S1). The real part of refractive index at the pump, both real parts at the probe frequency and all of the imaginary parts exhibit similar gradually decreasing tendencies for the permittivity and the index of refraction, whereas at the pump frequency the $\varepsilon_{real}(\omega_a)$ differs from $n(\omega_a)$ in pump dependence. Detuning of $n(\omega_a)$, $\varepsilon_{real}(\omega_e)$, n(ωe) and of $\varepsilon_{imag}(\omega_a)$ and $\kappa(\omega_a)$ gradually decreases with respect to the host by increasing the pump intensity. The pump dependent inversion effect on the real parts of the permittivity (except $\varepsilon_{real}(\omega_a)$) and refractive index detunes the resonance modes, thus proves that a numerical optimization is necessary.

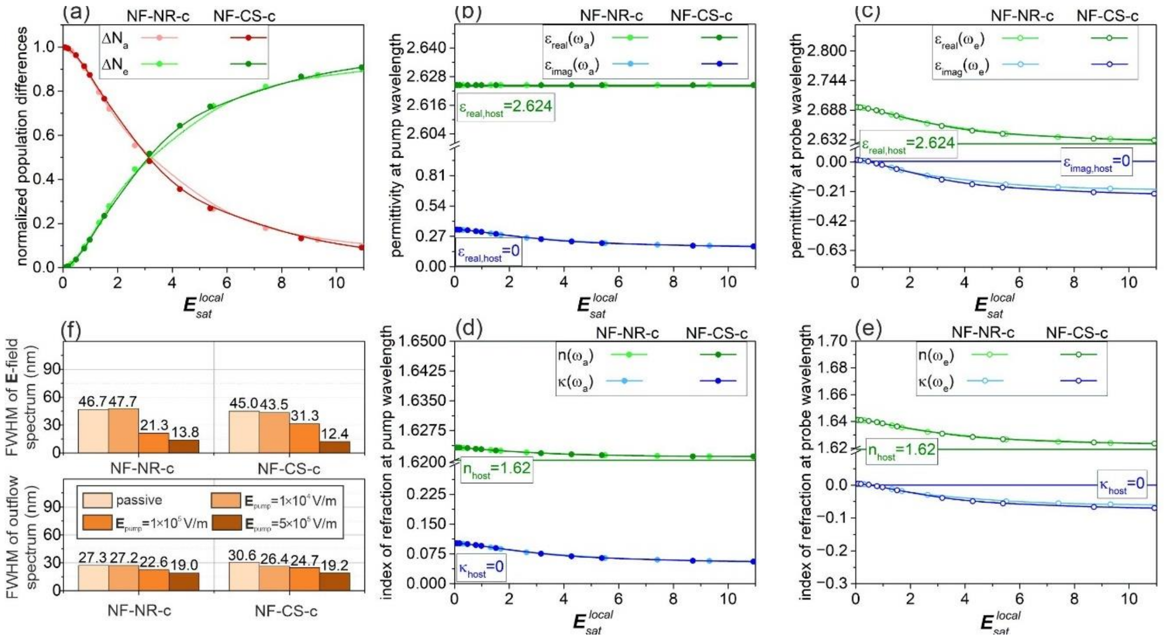


**Figure S1.** Pump dependent optical properties of optimized NF-c-type nanoresonators: (a) population differences, complex (b, c) dielectric permittivity and (d, e) index of refraction of the dye doped coating at the (b, d) pump and (c, e) probe wavelength, (f) line-width narrowing (decrease of the full-width-at-half-maximum (FWHM)) as a function of the pump intensity.

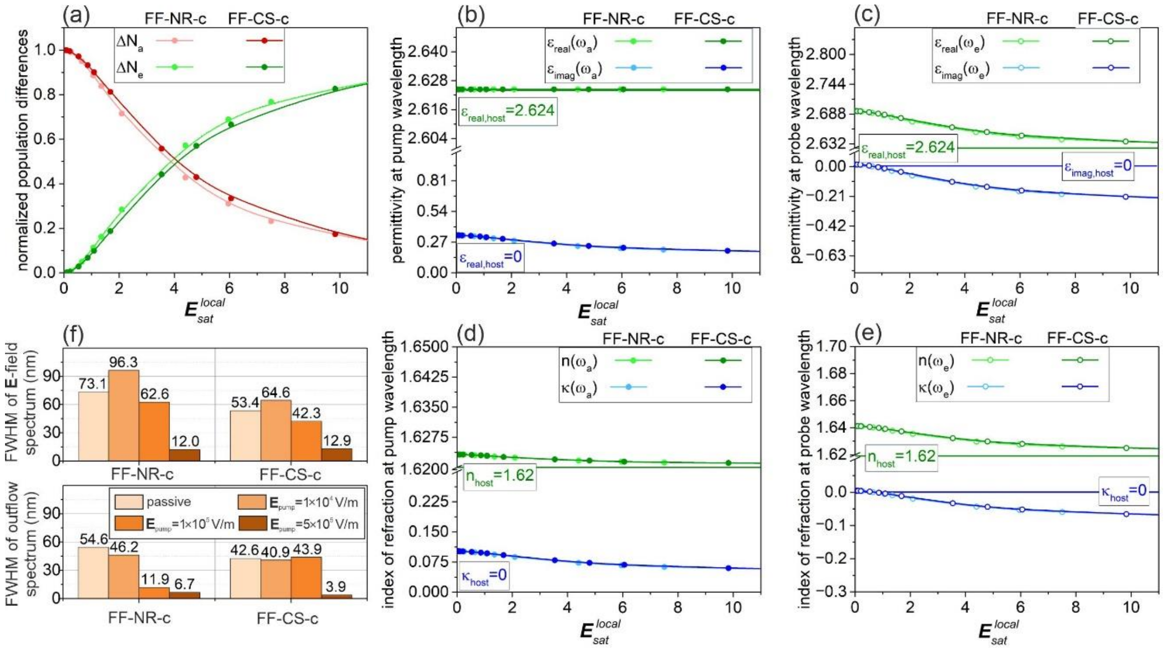


**Figure S2**. Pump dependent optical properties of optimized FF-c-type nanoresonators: (a) population differences, complex (b, c) dielectric permittivity and (d, e) index of refraction of the dye doped coating at (b, d) pump and (c, e) probe wavelength, (f) line-width narrowing (decrease of the full-width-at-half-maximum (FWHM)) as a function of the pump intensity.

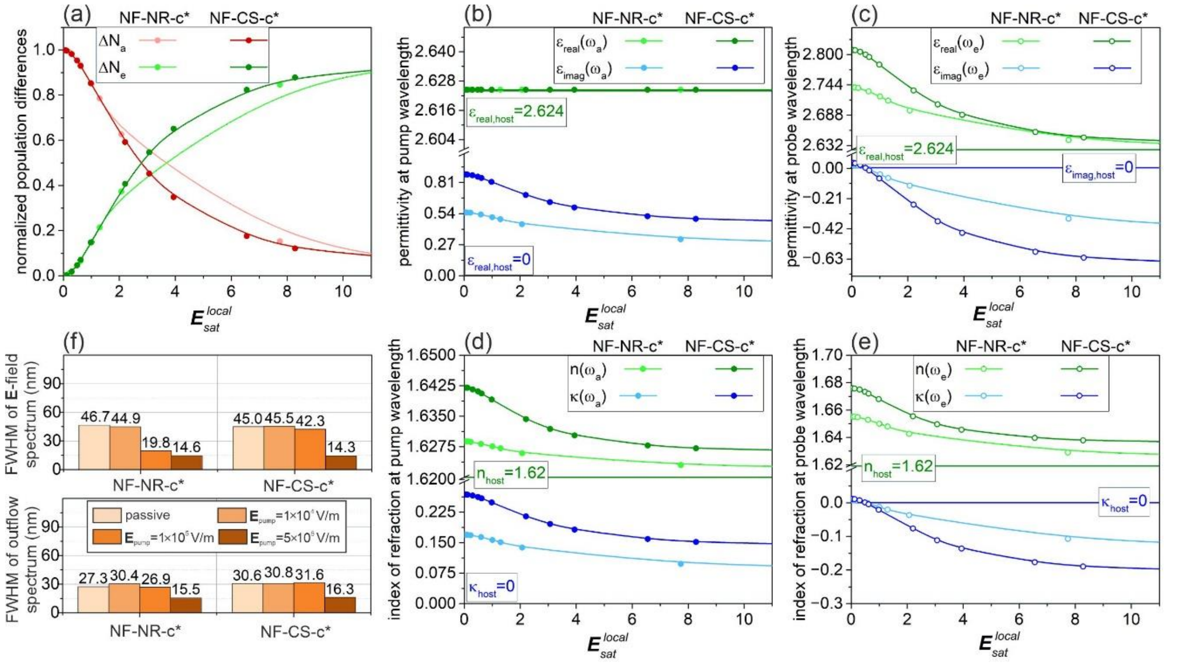


**Figure S3.** Pump dependent optical properties of post-optimization concentrated NF-c*-type nanoresonators: (a) population differences, complex (b, c) dielectric permittivity and (d, e) index of refraction of the dye doped coating at (b, d) pump and (c, e) probe wavelength, (f) line-width narrowing (decrease of the full-width-at-half-maximum (FWHM)) as a function of the pump intensity.

**Table S1.** Optical response of optimized and post-optimization concentrated systems. Geometry; $a_{long}$ & $a_{short}$: long and short full axis of nanorod; $r_{core}$ & $t_{shell}$: core radius and shell thickness of core-shell; AR: aspect ratio of rod ($a_{long}$/ $a_{short}$); GAR: generalized aspect ratio of core-shell ($r_{core}$/( $r_{core}$ + $t_{shell}$)). Optical response; gain: resistive heating in gain coating; outflow: power radiated into far-field; total: total incoming power of probe; ACS, SCS, ECS: absorption, scattering, extinction cross-sections; enhc.: enhancement with respect to incoming signal; avg: average; max: maximum; EQE: external quantum efficiency; IQE: internal quantum efficiency; (normalized by volume fraction ratio).

| | **NF-c** | | | | **FF-c** | | | | **NF-c*** | | | |
|---|---|---|---|---|---|---|---|---|---|---|---|---|
| | **NR** | | **CS** | | **NR** | | **CS** | | **NR** | | **CS** | |
| **geometry** | | | | | | | | | | | | |
| **$a_{long}$ & $r_{core}$ (nm)** | 59.38 | | 18.99 | | 97.33 | | 28.47 | | 59.38 | | 18.99 | |
| **$a_{short}$ & $t_{shell}$ (nm)** | 23.39 | | 5 | | 47.39 | | 8.85 | | 23.39 | | 5 | |
| **AR or GAR** | 2.54 | | 0.79 | | 2.05 | | 0.76 | | 2.54 | | 0.79 | |
| **gain coating (nm)** | 18.13 | | 24.62 | | 41.33 | | 47.45 | | 18.13 | | 24.62 | |
| **thresholds, peak and dip positions** | | | | | | | | | | | | |
| | $E_{sat}^{local}$ | $E_{sat}^{pump}$ | $E_{sat}^{local}$ | $E_{sat}^{pump}$ | $E_{sat}^{local}$ | $E_{sat}^{pump}$ | $E_{sat}^{local}$ | $E_{sat}^{pump}$ | $E_{sat}^{local}$ | $E_{sat}^{pump}$ | $E_{sat}^{local}$ | $E_{sat}^{pump}$ |
| **saturation** | 3.26 | 0.06 | 3.12 | 0.097 | 3.89 | 0.09 | 4.11 | 0.11 | 3.67 | 0.08 | 2.79 | 0.11 |
| **$\varepsilon_{imag}(\omega_e) < 0$** | 0.52 | 0.01 | 0.56 | 0.017 | 0.62 | 0.014 | 0.73 | 0.02 | 0.51 | 0.011 | 0.51 | 0.02 |
| **$\kappa(\omega_e) < 0$** | 0.52 | 0.01 | 0.56 | 0.017 | 0.62 | 0.015 | 0.73 | 0.02 | 0.51 | 0.011 | 0.51 | 0.02 |
| **lasing threshold** | 0.30 | 0.007 | 0.43 | 0.013 | 0.32 | 0.007 | 0.34 | 0.009 | 0.45 | 0.013 | 0.29 | 0.011 |
| **gain < 0** | 0.32 | 0.006 | 0.31 | 0.010 | 0.32 | 0.007 | 0.33 | 0.009 | 0.29 | 0.006 | 0.31 | 0.012 |
| **outflow > total** | - | - | - | - | 2.42 | 0.56 | 6.1 | 0.163 | 4.29 | 0.094 | 1.24 | 0.049 |
| **ACS < 0** | - | - | - | - | 2.48 | 0.057 | 5.77 | 0.154 | 4.24 | 0.092 | 1.26 | 0.05 |
| **ECS < 0** | - | - | - | - | - | - | - | - | 4.53 | 0.099 | 1.29 | 0.051 |
| **E-field peak** | - | - | - | - | - | - | - | - | 2.07 | 0.045 | 2.2 | 0.087 |
| **absorptance peak** | - | - | - | - | - | - | - | - | 2.07 | 0.045 | 2.2 | 0.087 |
| **SCS peak** | - | - | - | - | - | - | - | - | 2.07 | 0.045 | 2.2 | 0.087 |
| **ACS & ECS peaks** | 5.5 | 0.101 | - | - | - | - | 1.69 | 0.045 | 2.07 | 0.045 | 0.98 | 0.039 |
| **ACS & ECS dips** | - | - | - | - | - | - | - | - | 7.74 | 0.17 | 2.20 | 0.09 |
| **near-field properties and out-coupling efficiencies** | | | | | | | | | | | | |
| **pump enhc.** | 54 | | 32 | | 43.22 | | 37.36 | | 45.88 | | 25.36 | |
| **slope (avg)** | $2.16*10^5$ | | $9.8*10^4$ | | $6.83*10^4$ | | $4.5*10^4$ | | $4.84*10^5$ | | $1.87*10^5$ | |
| **slope (max)** | $8.52*10^5$ | | $4.97*10^5$ | | $3.57*10^5$ | | $2.9*10^5$ | | $1.88*10^6$ | | $8.83*10^5$ | |
| **max E-field (avg)** | $1.37*10^6$ | | $0.69*10^6$ | | $0.54*10^6$ | | $0.37*10^6$ | | $2.07*10^6$ | | $0.51*10^6$ | |
| **max E-field (max)** | $5.33*10^6$ | | $3.23*10^6$ | | $2.76*10^6$ | | $2.26*10^6$ | | $4.23*10^6$ | | $2.38*10^6$ | |
| **probe enhc. (avg)** | $1.37*10^2$ | | $0.69*10^2$ | | $0.54*10^2$ | | $0.37*10^2$ | | $2.07*10^2$ | | $0.51*10^2$ | |
| **probe enhc. (max)** | $5.33*10^2$ | | $3.23*10^2$ | | $2.76*10^2$ | | $2.26*10^2$ | | $4.23*10^2$ | | $2.38*10^2$ | |
| **quadrupolar ratio** | 1% (710 nm) | | 3% (710 nm) | | 1% (710 nm) | | 3% (710 nm) | | 5% (710 nm) (1% 700 & 720 nm) | | 3% (710 nm) (1% 705 & 720 nm) | |
| **EQE** | 0.88% | | 1.06% | | 0.32% (2.39%) | | 0.31% (1.42%) | | 4.04% | | 11.70% | |
| **IQE** | 0.88% | | 1.07% | | 0.32% (2.39%) | | 0.31% (1.42%) | | 4.47% | | 11.98% | |

| interval of complex permittivity and index of refraction | | | | | | |
|---|---|---|---|---|---|---|
| **$\varepsilon_{real}(\omega_a)$** | 2.62 | 2.62 | 2.62 | 2.62 | 2.62 | 2.62 |
| **$\varepsilon_{imag}(\omega_a)$** | [0.33; 0.17] | [0.33;0.18] | [0.33; 0.18] | [0.33;0.19] | [0.55; 0.29] | [0.88; 0.49] |
| **$n(\omega_a)$** | [1.623; 1.62] | [1.623;1.621] | [1.623; 1.62] | [1.623;1.621] | [1.629; 1.62] | [1.642; 1.627] |
| **$\kappa(\omega_a)$** | [0.1; 0.05] | [0.1;0.06] | [0.1; 0.06] | [0.1;0.06] | [0.17; 0.09] | [0.27; 0.15] |
| **$\varepsilon_{real}(\omega_e)$** | [2.69; 2.63] | [2.69;2.63] | [2.69; 2.63] | [2.69;2.63] | [2.74; 2.63] | [2.81; 2.65] |
| **$\varepsilon_{imag}(\omega_e)$** | [0.01; -0.21] | [0.01; -0.23] | [0.01; -0.24] | [0.01; -0.23] | [0.02; -0.41] | [0.04; -0.62] |
| **$n(\omega_e)$** | [1.64; 1.62] | [1.64;1.62] | [1.64; 1.623] | [1.64;1.62] | [1.66; 1.63] | [1.676; 1.638] |
| **$\kappa(\omega_e)$** | [0.004; -0.07] | [0.004; -0.07] | [0.004; -0.07] | [0.004; -0.07] | [0.007; -0.13] | [0.011; -0.189] |
| ***near-field spectrum:* linewidth and narrowing** | | | | | | |
| **passive FWHM** | 46.7 | 45 | 73.1 | 53.4 | 46.7 | 45 |
| **smallest FWHM** | 13.8 | 12.4 | 12 | 12.9 | 14.6 | 14.3 |
| **degree of narrowing** | 3.4 | 3.6 | 6.1 | 4.1 | 3.2 | 3.1 |
| ***far-field spectrum:* linewidth and narrowing** | | | | | | |
| **passive FWHM** | 27.3 | 30.6 | 54.6 | 42.6 | 27.3 | 30.6 |
| **smallest FWHM** | 19.0 | 19.2 | 6.7 | 3.9 | 15.5 | 16.3 |
| **degree of narrowing** | 1.4 | 1.6 | 8.2 | 10.9 | 1.8 | 1.9 |
